# Arthroscopic Rotator Cuff Repair Improves Sleep Disturbance and Quality of Life: A Prospective Study

**DOI:** 10.3390/ijerph18073797

**Published:** 2021-04-06

**Authors:** Umile Giuseppe Longo, Vincenzo Candela, Sergio De Salvatore, Ilaria Piergentili, Nicolò Panattoni, Erica Casciani, Aurora Faldetta, Anna Marchetti, Maria Grazia De Marinis, Vincenzo Denaro

**Affiliations:** 1 Department of Orthopaedic and Trauma Surgery, Campus Bio-Medico University, Via Alvaro del Portillo, 200, Trigoria, 00128 Rome, Italy; v.candela@unicampus.it (V.C.); s.desalvatore@unicampus.it (S.D.S.); ilaria.piergentili94@gmail.com (I.P.); casciani15@gmail.com (E.C.); denaro@unicampus.it (V.D.); 2 Department of Biomedicine and Prevention, University of Rome Tor Vergata, 00133 Rome, Italy; nicolo.panattoni@alumni.uniroma2.eu; 3 Research Unit Nursing Science, Campus Bio-Medico di Roma University, 00128 Rome, Italy; faldetta.cbm@gmail.com (A.F.); a.marchetti@unicampus.it (A.M.); m.demarinis@unicampus.it (M.G.D.M.)

**Keywords:** Pittsburgh Sleep Quality Index (PSQI), sleep quality, sleep disturbance, rotator cuff tear, rotator cuff repair, arthroscopy, shoulder, surgery

## Abstract

Sleep disturbances are very common in patients with rotator cuff injury. Improvement of sleep quality in these patients can be considered a significant factor for healing in conjunction with surgery. The primary objective of this prospective study was to evaluate changes in sleep quality after surgery in patients with rotator cuff repair by analyzing the PSQI (Pittsburgh Sleep Quality Index) score. The secondary aim was to evaluate the improvement in quality of life in terms of functional limitations and shoulder pain after surgery. Fifty-eight patients with rotator cuff tears treated by arthroscopic surgery were included. All the patients completed the PSQI, the 36-Item Short Form Survey (SF-36), the Simple Shoulder Test (SST), the American Shoulder and Elbow Surgeons Shoulder Score (ASES), the Oxford Shoulder Score (OSS) and the Constant-Murley Score (Constant) before and at one, three and six months after surgery. Overall improvement in all the scores analyzed (*p* < 0.001) was found. Preoperative and postoperative PSQI scores correlated with SF-36, SST, ASES and Constant scores at each follow-up. Preoperative and one-month postoperative OSS correlated with the PSQI score. Using the Friedman test, we found an overall improvement in all score analyses (*p* < 0.001). The results prove that after rotator cuff repair, sleep disturbances improve three to six months after surgery improving the quality of life of these patients.

## 1. Introduction

Rotator cuff injury is the cause of approximately 70% of all outpatient visits for shoulder pain. Rotator cuff tear is found in 20% to 54% of people between the ages of 60 and 80 [1]. This injury is often the result of a degenerative process of the shoulder as in the normal ageing of a person and could be asymptomatic and difficult to recognize early. However, it can also be a symptomatic and very disabling alteration secondary to a traumatic event or a shoulder dislocation [2]. Although in some conditions rotator cuff tears are irreparable, in general, arthroscopic repair significantly improves the quality of life [3]. As reported in the literature, the socioeconomic impact of rotator cuff tear repair in the working population is continuously growing [4].

Sleep disturbances are among the most frequent health disorders with a prevalence of 15% to 20% in the general adult population [5]. Sleeping has important consequences on biological functions, physiological recovery, learning, memory, cognitive processes and emotional health [6]. In rotator cuff-injured patients, sleep disturbances are very common in the preoperative period with a prevalence of 89% [7]. Frequently, sleep disturbance is the leading cause that addresses the patient to surgery. In most cases, patients’ complaints are about night pain which causes sleep disturbances in the short term (such as insomnia and lack of sleep) rather than the loss of shoulder function. The quality of sleep is affected in particular by shoulder pain and by the prolonged forced position taken during the night [8]. The etiology of pain is due to increased levels of proinflammatory factors and pain-related cytokines [8].

Therefore, the improvement of sleep quality in patients with rotator cuff tear can be considered a significant factor for healing in conjunction with surgery [3,6]. Despite this fundamental role of sleep quality in the recovery of these patients, there are few studies on this topic in the literature.

The primary objective of this paper was to evaluate changes in sleep quality after surgery in patients with rotator cuff arthroscopic repair by analysing the PSQI (Pittsburgh Sleep Quality Index) score. The secondary aim was to evaluate improvement in the quality of life in terms of functional limitations and shoulder pain after surgery.

## 2. Materials and Methods

This article was written in accordance with the STROBE (Strengthening the Reporting of Observational Studies in Epidemiology) guidelines to improve the quality of reporting of this observational study. Shoulder surgeries were performed in 104 patients of the Department of Orthopedic and Traumatological Surgery of our University Hospital. This prospective study included patients with rotator cuff tear undergoing rotator cuff arthroscopic repair (RCR). The tears were assessed by preoperative magnetic resonance imaging (MRI) and clinical examination by two orthopedic surgeons specializing in shoulder arthroscopy. Only patients with Goutallier grade 2 [9] and Patte stage 2 lesions [10] were included in our study. All the patients had previously been conservatively treated with physical therapy involving a rehabilitation program and corticosteroid injections. The arthroscopic repair included single- and double-bundle techniques [11,12]. All the procedures were performed by the same senior surgeon. Patients not undergoing surgery or other types of shoulder pathologies were excluded. All the patients completed a standardized rehabilitation protocol [13]. During the first four weeks, the arm was supported with an abduction sling pillow, and pendulum exercises, table slide, and active elbow extension and flexion were allowed. Small circular pendulum exercises were performed. For the table slide exercise, the patient slides the hand of the operated shoulder forward on a surface by advancing the chest towards the table. The patients started therapy four weeks after surgery, working with the therapist 1–3 times/week and at home on the other days. From week 5 to week 8, the patients performed passive forward elevation, passive external rotation, and from week 5, active assisted range of motion (ROM) to tolerance. From week 8 to week 10, the patients progressed towards active ROM to tolerance. After week 10, the patients started the rehabilitation of the deltoid, scapular stabilizers and the rotator cuff with concentric and eccentric exercise strengthening. The study was conducted between January 2019 and September 2019. Patients with at least six months of follow-up were included. Of the 104 patients eligible for this study, only 58 completed the questionnaires at all follow-up timepoints and were included in the study. The age of the patients ranged between 18 and 87 years, and there were 29 (50%) women and 29 (50%) men.

The PSQI is a 19-question survey used to measure through self-assessment the quality of sleep in patients [14]. It is usually used to monitor sleep disturbance over time. The 19 self-rated questions assess a wide variety of factors relating to sleep quality, including estimates of sleep duration and latency and of the frequency and severity of specific sleep-related problems [14]. To assess overall sleep quality, seven sleep domains (sleep quality, sleep latency, sleep duration, habitual sleep efficiency, sleep disturbances, sleep medication use and daytime dysfunction) are analyzed and evaluated by the questionnaire [15]. In scoring the PSQI, seven component scores are derived, each scored from 0 (no difficulty) to 3 (severe difficulty). The component scores are summed to produce a global score (ranging from 0 to 21). Lower scores indicate a better quality of sleep [14].

The study participants completed the PSQI [15], the 36-Item Short Form Survey (SF-36) [16], the Simple Shoulder Test (SST) [17], the American Shoulder and Elbow Surgeons Shoulder Score (ASES) [18], the Oxford Shoulder Score (OSS) [19] and the Constant-Murley score before surgery and at one, three and six months after surgery. The PSQI score has a possible range from a minimum of 0 to a maximum of 21 points. Lower scores in the PSQI indicate a better outcome. On the contrary, better outcomes of SF-36, SST, ASES, OSS and Constant scores are indicated with higher scores.

All the data were analyzed using IBM SPSS Statistics for Windows, Version 26.0. (Armonk, NY; IBM Corp). To assess data normality, the Shapiro–Wilk test was used. Since the data did not respect the normality distribution, baseline and postoperative follow-up scores were compared using the Friedman test and the Wilcoxon signed-rank test. The numerical data were expressed with the means and standard deviations. Correlation analysis using the Spearman’s rho coefficient was performed between the PSQI score and SF-36, SST, ASES, OSS and Constant scores at each follow-up. Two-tailed *p*-values of < 0.05 were considered statistically significant.

An a priori power analysis was conducted using G*Power 3.1. With an alpha level of 0.05, minimum power established at 0.80 and a medium effect size of 0.491 [20], 35 participants would be necessary to find a statistically significant effect.

## 3. Results

This study included a total of 58 patients (29 females and 29 males) with an average age of 63.4 ± 13 years (ranging between 18 and 87 years).

Regarding multivariate change, using the Friedman test, we found overall improvement in the PSQI (*p* < 0.001), SF-36 (*p* < 0.001), SST (*p* < 0.001), ASES (*p* < 0.001), OSS (*p* < 0.001) and Constant scores (*p* < 0.001) in the patients who underwent arthroscopic repair (Table 1).

**Figure 1:**
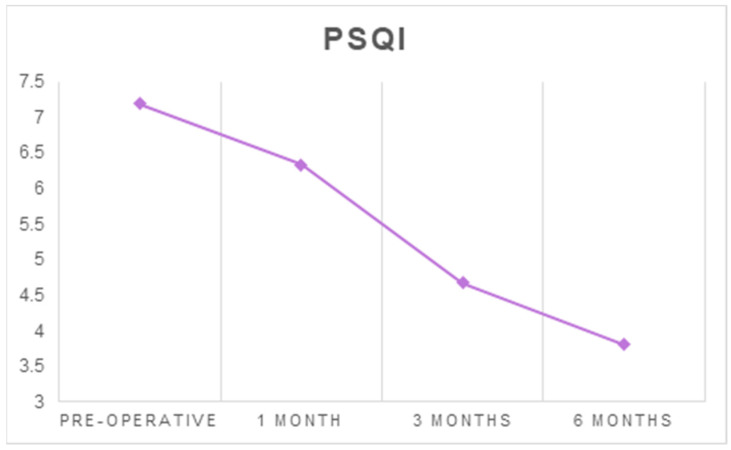
The average scores of the PSQI at each follow-up timepoint.

The mean PSQI score decreased from 7.19 ± 3.91 before surgery to 3.81 ± 3.32 at six months after surgery (*p* < 0.001) (Table 1 and Table 2, Figure 1).

**Figure 2:**
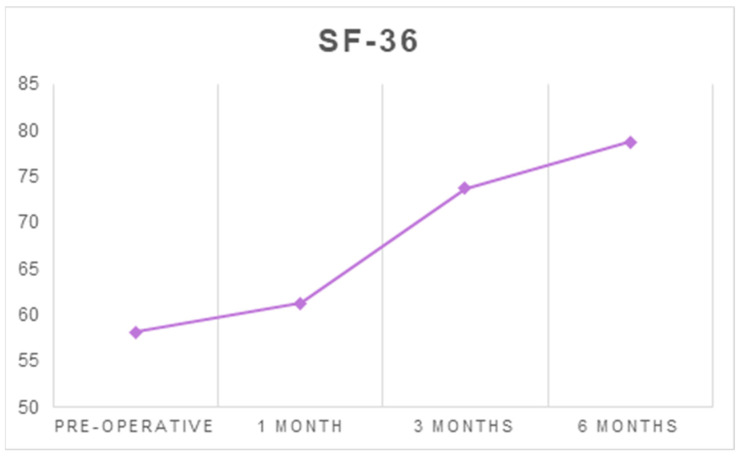
The average scores of SF-36 at each follow-up timepoint.

The mean SF-36 score increased from 58.15 ± 17.35 before surgery to 78.75 ± 14.9 at six months after surgery (*p* < 0.001) (Table 1 and Table 2, Figure 2).

**Figure 3:**
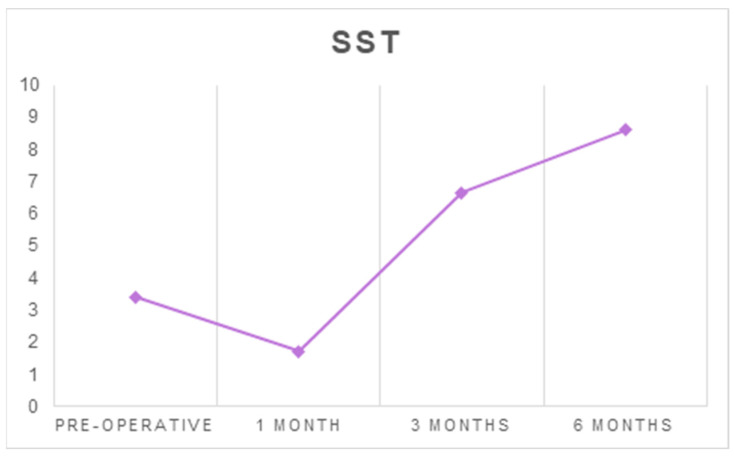
The average scores of SST at each follow-up timepoint.

The mean SST score increased from 3.41 ± 2.73 before surgery to 8.62 ± 2.65 at six months after surgery (*p* < 0.001) (Table 1 and Table 2, Figure 3).

**Figure 4:**
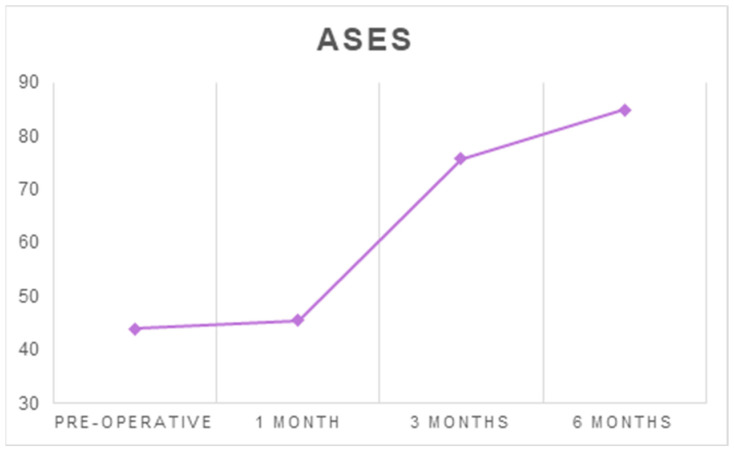
The average ASES at each follow-up timepoint.

The mean ASES increased from 43.94 ± 20.4 before surgery to 84.95 ± 13.27 at six months after surgery (*p* < 0.001) (Table 1 and Table 2, Figure 4).

**Figure 5:**
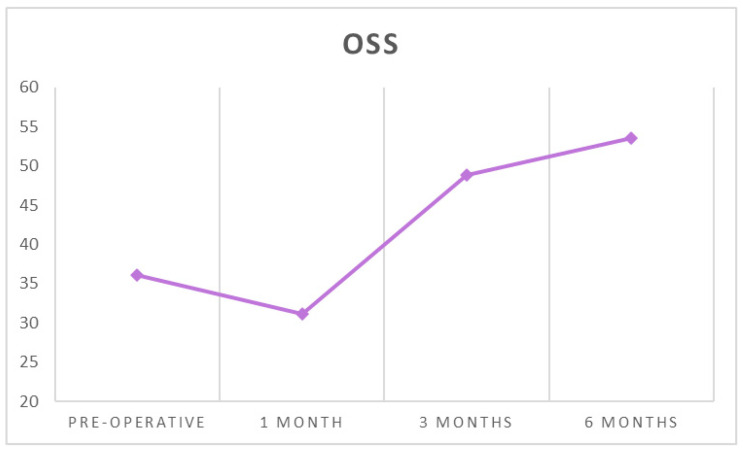
The average OSS at each follow-up timepoint.

The mean OSS increased from 36.09 ± 10.96 before surgery to 53.60 ± 5.79 at six months after surgery (*p* < 0.001) (Table 1 and Table 2, Figure 5).

**Figure 6:**
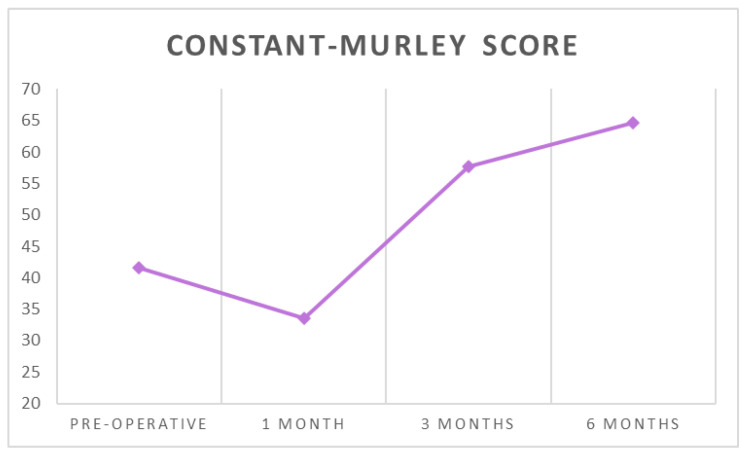
The average Constant scores at each follow-up timepoint.

The mean Constant score increased from 41.6 ± 15.58 before surgery to 64.59 ± 9.85 at six months after surgery (*p* < 0.001) (Table 1 and Table 2, Figure 6).

Only for the PSQI and ASES between preoperative time and one-month postoperatively, no statistically significant differences were found (*p* = 0.098 and *p* = 0.533, respectively) (Table 2).

Preoperative and postoperative PSQI scores correlated with SF-36, SST, ASES and Constant scores at each follow-up timepoint, while they correlated with the OSS only at one-month postoperatively (Table 3). At each follow-up timepoint, as the PSQI score decreased, SF-36, SST, ASES and Constant scores increased. At one-month postoperatively, as the PSQI decreased, the OSS increased.

## 4. Discussion

The present study assessed different dimensions of sleep quality using a validated and reliable assessment tool, the PSQI, in a sample of 58 consecutive patients undergoing rotator cuff tear repair surgery. The results showed that sleep quality and quality of life increased with surgery, while functional limitations and the pain decreased. In the preoperative period, poor sleep quality was reported in all the patients included with the mean PSQI value of 7.19. In general, the PSQI progressively improved across the various follow-up timepoints, reaching the best value at the last follow-up timepoint.

Horneff et al. [21] reported similar results in a case series study demonstrating that sleep disorders were common in the rotator cuff tear population, mentioned by 89% of patients included before surgery. More than 62% of these patients showed statistically significant improvement from the third month of follow-up after surgery, with an improvement trend of up to two years.

Austin et al. [22] analysed the use of narcotics related to sleep disorders. Prolonged use of preoperative and postoperative narcotics is related to worsening of sleep disturbances following repair surgery and therefore patients are advised to discontinue narcotics before surgery and as soon as possible after surgery [22].

Maestroni et al. [23] reported that the etiology of the worsening in sleep quality is multifactorial, showing that patients’ pain perception contributes to insomnia. This study aimed to evaluate which factor was associated with higher pain and disability in rotator cuff-injured patients. Their findings suggest that patients with rotator cuff tears should be evaluated carefully, as pathologic findings could not correspond to perceived pain and disability.

Other studies state how common nocturnal shoulder pain is present in patients with a rotator cuff tear. In particular, Khazzam et al. [6] described specific factors that correlated with poor sleep quality, such as female gender, depression, presence of low back pain, diabetes mellitus, cervical involvement and a high body mass index (BMI). Therefore, rotator cuff tears should not be the cause of insomnia, and it is important to distinguish the real origin of the sleep disturbance.

Werner et al. [24] investigated the negative influence of subacromial pressure in four positions assumed during sleep (supine, prone, lateral decubitus, supine with abducted shoulders) and postoperative healing of rotator cuff repair. Tendon perfusion is crucial for postoperative tendon–bone healing, and Werner et al. concluded that supine position is the most adequate to ensure a good recovery. At the same time, it is therefore recommended to avoid the lateral position on the injured shoulder in order to reduce the subacromial pressure. Holdaway et al. [25] performed a cross-sectional analysis comparing sleep positions and shoulder pain. They found that a priori high-risk sleep positions (free fall and starfish) revealed a protective association.

Gumina et al. [26] differentiate sleep disturbances in relation to the size of the rotator cuff tear. Patients with small tears have worse sleep quality than those with a severe tear in terms of longer falling asleep and frequent awakenings. This may be due to the greater amount of inflammatory tissue usually associated with small tears compared to that seen in large, massive lesions. Visual analog scale scores (VAS) are higher in patients with smaller rotator cuff tears.

The second aim of this study was to assess the quality of life improvement after surgery. Patients reported an improvement in functionality, pain and performance in daily living activities starting from three months after surgery. The improvements remained stable during six months of follow-up.

A limitation of this study is lack of a control group treated conservatively and the lack of follow-up longer than six months. Patients included in this study reported only Goutallier grade 2 and Patte stage 2 lesions. The results of this study were related to these types of lesions; therefore, it was not possible to define precise results in other types of RCTs. The severity of the patient’s condition affects the outcome; therefore, further randomized controlled studies including other types of lesions are required to confirm our results. The strength of this study is due to the poor evidence in the literature on the correlation between sleep quality improvement and surgery. This study aimed to reinforce the scientific evidence regarding the hypothesis that sleep disturbances were common in patients with rotator cuff tears and sleep quality improved after surgical repair.

## 5. Conclusions

The results of this study support the hypothesis that after rotator cuff repair, sleep disturbances improve three to six months after surgery improving the quality of life of this patient population. Future randomized controlled trials with longer follow-up aimed to assess sleep quality in the rotator cuff tear population treated conservatively and surgically are required.

## Figures and Tables

**Table 1:** Statistical follow-up analysis (mean ± SD). Note: * = statistically significant.

	Preoperative	One Month	Three Months	Six Months	*p*-Value
PSQI	7.19 ± 3.91	6.34 ± 4.01	4.67 ± 3.31	3.81 ± 3.32	<0.001 *
SF-36	58.15 ± 17.35	61.26 ± 14.69	73.68 ± 15.01	78.75 ± 14.9	<0.001 *
SST	3.41 ± 2.73	1.71 ± 1.97	6.67 ± 2.21	8.62 ± 2.65	<0.001 *
ASES	43.94 ± 20.4	45.52 ± 16.92	75.74 ± 17.09	84.95 ± 13.27	<0.001 *
OSS	36.09 ± 10.96	31.09 ± 8.18	48.88 ± 7.72	53.60 ± 5.79	<0.001 *
Constant score	41.6 ± 15.58	33.56 ± 10.46	57.66 ± 11.87	64.59 ± 9.85	<0.001 *

**Table 2:** Score differences in the follow-up periods. Note: * = statistically significant.

	Pre–One Month	Pre–Three Months	Pre–Six Months	One Month–Three Months	One Month–Six Months	Three Months–Six Months
PSQI	0.098	<0.001 *	<0.001 *	<0.001 *	<0.001 *	<0.001 *
SF-36	0.05 *	<0.001 *	<0.001 *	<0.001 *	<0.001 *	0.01 *
SST	<0.001 *	<0.001 *	<0.001 *	<0.001 *	<0.001 *	<0.001 *
ASES	0.533	<0.001 *	<0.001 *	<0.001 *	<0.001 *	<0.001 *
OSS	0.02 *	<0.001 *	<0.001 *	<0.001 *	<0.001 *	<0.001 *
Constant score	0.002 *	<0.001 *	<0.001 *	<0.001 *	<0.001 *	<0.001 *

**Table 3:** Correlation between the PSQI and SF-36, SST, ASES and OSS. Note: (*) = statistically significant.

		Preoperative PSQI	PSQI, One Month	PSQI, Three Months	PSQI, Six Months
**SF-36**	rho	−0.570	−0.594	−0.577	−0.538
	*p*-value	<0.001 *	<0.001 *	<0.001 *	<0.001 *
**SST**	rho	−0.585	−0.604	−0.359	−0.498
	*p*-value	<0.001 *	< 0.001 *	0.006 *	<0.001 *
**ASES**	rho	−0.505	−0.544	−0.547	−0.393
	*p*-value	<0.001 *	<0.001 *	<0.001 *	0.002 *
**OSS**	rho	−0.071	−0.317	−0.136	−0.195
	*p*-value	0.598	0.015 *	0.308	0.142
**Constant score**	rho	−0.527	−0.504	−0.302	−0.383
	*p*-value	< 0.001	<0.001	0.021	0.003

## Data Availability

The data presented in this study are available on request from the corresponding author. The data are not publicly available due to privacy.
